# Dual Sensory Loss and Rate of Dementia Symptom Progression in Cognitively Normal Older Adults

**DOI:** 10.1093/geroni/igaf122.3574

**Published:** 2025-12-31

**Authors:** Kathryn Hand, Lilah Besser, Walter Kukull, Willa Brenowitz

**Affiliations:** Oregon Health and Science University, Portland, Oregon, United States; University of Miami, Boca Raton, Florida, United States; University of Washington, Seattle, Washington, United States; Kaiser Permanente, Portland, Oregon, United States

## Abstract

Loss of both hearing and vision may serve as a stronger predictor of the rate of dementia symptom progression (i.e., clinical progression) than loss of either sense individually. We evaluated the longitudinal associations of hearing, vision, and dual sensory loss with clinical progression among 21,098 participants in the National Alzheimer’s Coordinating Center’s Uniform Data Set, restricting to those who were cognitively normal at baseline. Participants were 69 ±11 years at baseline, and followed annually for an average of 3.7 years, 66% were female, 78% White, 2.9% visually impaired, 18.6% hearing impaired, and 1.6% dual sensory impaired. Participants were rated by clinicians as vision impaired if they had trouble with vision after correction and as hearing impaired if they had trouble with hearing without any correction. Dementia staging was measured using the Clinical Dementia Rating Sum of Boxes (CDRSUM). Linear mixed-effects models estimated associations, adjusting for age, sex, race, education, cardiovascular disease, diabetes, hypertension, depression, and APOE-ε4 status. At baseline, sensory impairment was not significantly associated with CDRSUM; however, significant interactions with time were observed. Compared to those with no sensory impairments, individuals with dual impairment had the highest annual increase in CDRSUM (β = 0.075, 95%CI:0.048–0.102), followed by those with vision impairment alone (β = 0.028, 95%CI:0.006–0.049) and hearing impairment alone (β = 0.019, 95%CI:0.011–0.028). Dual sensory impairment (versus single sensory impairment) is associated with greater clinical progression of dementia symptoms over time, highlighting the potential importance of early identification and management of dual sensory loss.

